# P-528. Evaluation of Saliva-Based cCMV Testing at the University of Rochester Medical Center (URMC) and Hearing Outcomes of Infected Infants

**DOI:** 10.1093/ofid/ofaf695.743

**Published:** 2026-01-11

**Authors:** Iris Yau, Mike Sportiello, Geoffrey A Weinberg, Mary T Caserta, Christina M Ashrafioun, Jennifer L Nayak

**Affiliations:** University of Rochester School of Medicine and Dentistry, Rochester, NY; Emory University, Atlanta, GA; University of Rochester Sch Med & Dent, Rochester, New York; University of Rochester Medical Center, Rochester, New York; University of Rochester, Rochester, New York; University of Rochester Medical Center, Rochester, New York

## Abstract

**Background:**

Congenital cytomegalovirus (cCMV) affects approximately 1 in 200 neonates and can lead to severe disease in the newborn period. Sensorineural hearing loss (SNHL) and developmental delays are the most common long-term sequelae. Prior to the implementation of a 1 year cCMV universal screening program in New York State (NYS), there was a state-mandated hearing-targeted cCMV screening program utilizing saliva-based PCR testing. All positive salivary cCMV PCR tests were confirmed by a urine PCR due to possible false-positive results from contamination of saliva by maternal breast milk containing CMV. This study aims to explore the performance characteristics of salivary cCMV PCR testing at URMC.

**Methods:**

A retrospective chart review of neonates who underwent saliva-based CMV PCR testing at URMC hospitals between March 1, 2019 and October 31, 2023 was completed. The primary outcomes were the false-positive rate and positive predictive value (PPV) of saliva-based CMV testing. The secondary outcome was the rate of SNHL among infants with confirmed cCMV infection through annual audiologic follow up.

**Results:**

Out of 37 charts reviewed, 26 patients had both saliva and urine testing performed. Of the 26 neonates, 12 (46%) with positive salivary CMV PCR tests were confirmed positive by urine PCR testing; 14 of the 26 neonates (54%) had false positive salivary CMV PCR results. The PPV of saliva-based CMV testing was 46%. Of the 19 infants with a positive urine test during this period, 7 (36%) had moderate to profound hearing loss. Eight patients were lost to audiologic follow-up during the study period.

**Conclusion:**

The PPV of 46% confirms that while salivary testing may be a useful initial screening test due to the ease of sample collection, confirmation by urine PCR testing is necessary. Although hearing-targeted cCMV newborn screening is able to identify newborns with cCMV and early onset SNHL, it fails to identify those with cCMV who pass the hearing screen at birth but remain at high risk for hearing loss. Ongoing efforts to evaluate universal cCMV screening in NYS may decrease this limitation, potentially enabling a larger population of children to benefit from earlier intervention.

**Disclosures:**

Geoffrey A. Weinberg, MD, Inhalon Biopharma: Advisor/Consultant|Merck & Co: Honoraria Mary T. Caserta, MD, Merck: Grant/Research Support|Moderna: Grant/Research Support Jennifer L. Nayak, MD, Merck: Grant/Research Support|Moderna: Grant/Research Support|Pfizer, Inc.: Grant/Research Support|Sanofi: Grant/Research Support

